# Using integrated analysis from multicentre studies to identify RNA methylation-related lncRNA risk stratification systems for glioma

**DOI:** 10.1186/s12935-023-03001-w

**Published:** 2023-08-05

**Authors:** Fanxuan Huang, Xinyu Wang, Junzhe Zhong, Hao Chen, Dan Song, Tianye Xu, Kaifu Tian, Penggang Sun, Nan Sun, Jie Qin, Yu Song, Wenbin Ma, Yuxiang Liu, Daohan Yu, Xiangqi Meng, Chuanlu Jiang, Hanwen Xuan, Da Qian, Jinquan Cai

**Affiliations:** 1 Department of Neurosurgery, The Second Affiliated Hospital of Harbin Medical University, No. 246, Xuefu Road, Nangang District, Harbin, 150086 China; 2 Future Medical Laboratory, The Second Affiliated Hospital of Harbin Medical University, Harbin, 150086 China; 3 Department of Burn and Plastic Surgery-Hand Surgery, Changshu Hospital Affiliated to Soochow University, Changshu No. 1 People’s Hospital, Changshu, 215500 Jiangsu Province China

**Keywords:** Chemotherapy, Glioma, Immunotherapy, LncRNAs, Prognosis, RNA methylation

## Abstract

**Background:**

*N*6-methyladenosine (m6A), 5-methylcytosine (m5C) and *N*1-methyladenosine (m1A) are the main RNA methylation modifications involved in the progression of cancer. However, it is still unclear whether RNA methylation-related long noncoding RNAs (lncRNAs) affect the prognosis of glioma.

**Methods:**

We summarized 32 m6A/m5C/m1A-related genes and downloaded RNA-seq data and clinical information from The Cancer Genome Atlas (TCGA) database. Differential expression analysis and weighted gene co-expression network analysis (WGCNA) were used to identify differentially expressed (DE-) RNA methylation-related lncRNAs in order to construct a prognostic signature of glioma and in order to determine their correlation with immune function, immune therapy and drug sensitivity. In vitro and in vivo assays were performed to elucidate the effects of RNA methylation-related lncRNAs on glioma.

**Results:**

A total of ten RNA methylation-related lncRNAs were used to construct a survival and prognosis signature, which had good independent prediction ability for patients. It was found that the high-risk group had worse overall survival (OS) than the low-risk group in all cohorts. In addition, the risk group informed the immune function, immunotherapy response and drug sensitivity of patients with glioma in different subgroups. Knockdown of RP11-98I9.4 and RP11-752G15.8 induced a more invasive phenotype, accelerated cell growth and apparent resistance to temozolomide (TMZ) both in vitro and in vivo. We observed significantly elevated global RNA m5C and m6A levels in glioma cells.

**Conclusion:**

Our study determined the prognostic implication of RNA methylation-related lncRNAs in gliomas, established an RNA methylation-related lncRNA prognostic model, and elucidated that RP11-98I9.4 and RP11-752G15.8 could suppress glioma proliferation, migration and TMZ resistance. In the future, these RNA methylation-related lncRNAs may become a new choice for immunotherapy of glioma.

**Supplementary Information:**

The online version contains supplementary material available at 10.1186/s12935-023-03001-w.

## Introduction

Glioma is the most common and aggressive primary brain tumour [1, 2]. Surgery combined with temozolomide (TMZ) chemotherapy and radiotherapy to the lesion area are currently commonly used in clinical treatment. Although scientists have made some progress in both clinical and basic research and comprehensive treatment in the past few decades, the overall cure rate for gliomas, especially glioblastomas (GBMs), remains poor, and affected patients have very poor prognoses [3, 4]. Several factors contribute to glioma progression and patient survival, including the location of the tumour, the patient's age, mutations in cancer cells, and epigenetic dysregulation [5, 6]. Therefore, further investigation of the mechanisms of glioma occurrence and development, as well as the development of new treatment strategies, are urgently needed.

LncRNA molecules have a conserved secondary structure and can be as long as 200 nucleotides; however, they do not encode any proteins [5, 7]. It was initially discovered that lncRNAs were involved in epigenetic regulation during embryogenesis involving the inactivation of the X chromosome [8]. Over the past few years, there has been a steadily increasing interest in identifying lncRNAs and understanding how they regulate almost all aspects of gene expression, protein translation, and stability, including both cis (regulates neighbouring genes) and trans (regulates distant genes) [9]. To regulate gene expression, small RNAs engage with proteins, DNA, and RNA through a variety of molecular mechanisms, such as chromatin remodelling, mRNA degradation, splicing, and translational control [10, 11]. Increasing numbers of lncRNAs have been discovered to play an essential role in the epigenetic regulation of tumours, suggesting that they could serve as biomarkers and therapeutic targets to provide patients with more effective treatment, diagnosis, and prognosis. The lncRNA ADAMTS9-AS2 was reported to recruit DNMT1/3 to the CDH3 promoter, inhibiting oesophageal cancer cell proliferation, invasion, and migration [12]. Through competitive binding to miR-20b-3p and activation of the Stat3/p300 complex, lnc-TALC has been shown to influence the c-Met signalling pathway by promoting the expression of the DNA repair enzyme O6-methylguanine-DNA methyltransferase (MGMT) as well as resistance to TMZ [13].

An epigenetic modification known as methylation regulates the stability and expression of genetic material in cells, and it is catalysed by methyltransferases (‘writers’), which utilize S-adenosylmethionine (SAM) as a methyl donor. In addition to writers, there are dedicated ‘erasers’ (demethylases) and methyl ‘readers’ [14–16]. Studies have shown that the methylation of DNA and RNA is involved in the regulation of various tumour-related pathways [17, 18]. Methylation is the most common RNA modification found in eukaryotes [14, 19]. More than 150 types of RNA methylation have been identified, the most common of which is *N*6-methyladenosine (m6A), 5-methylcytosine (m5C), *N*1-methyladenosine (m1A), and 5-hydroxymethylcytosine (5hmC) [16]. An imbalance in the methylation of RNA is one of the main characteristics of most malignant tumours. By increasing the expression of the mTORC1 adaptor protein WTAP, mTORC1 signalling induces m6A methyltransferase and induces the modification of m6A RNA, which promotes cell growth [20]. NSUN2 and TRDMT1 are two m5 C methylases that have been closely associated with cell cycle control in both cancer and stem cell biology [21]. However, there is no evidence to explain the association between RNA methylation-related lncRNAs and glioma.

In this study, we determined the prognostic implication of RNA methylation-related lncRNAs in glioma and established an RNA methylation-related lncRNA prognostic signature, which might provide insights into prognosis evaluation and convenience for decision-making in clinical practice. Finally, we examined the effects of RNA methylation-related lncRNAs knocked down on glioma proliferation, invasion, TMZ chemotherapy and global methylation level. Our results offer novel insights into the functional role of RNA methylation-related lncRNAs in glioma.

## Materials and methods

### Data source

The glioma specimens (n = 4) and normal brain tissue samples (n = 4) were taken from glioma patients who underwent partial resection of tumour-adjacent tissue (Department of Neurosurgery, The Second Affiliated Hospital of Harbin Medical University, Harbin, China). The experiment has passed ethical review by the medical ethics committee of The Second Affiliated Hospital of Harbin Medical University. In addition to transcriptomic data related to glioma, clinic-related information was also obtained from the freely accessible TCGA (https://portal.gdc.cancer.gov/) and CGGA databases (http://www.cgga.org.cn/). As part of the TCGA database, transcriptomic data are available from 675 samples, three of which are normal and 672 of which are glioma samples (511 LGG and 161 GBM samples). A comprehensive analysis of the survival data for 667 of the 672 glioma samples was performed to identify prognostic genes and to assess the effectiveness of prognostic signatures. As an external validation cohort, the CGGA database contains transcriptomic data for 693 glioma samples, 443 of which are LGG samples and 249 of which are GBM samples.

### Collection of RNA methylation regulators

The latest literature review identified 32 known m6A, m5C, and m1A RNA methylated modulators that have been reported in the literature during the past couple of years. There were six m1A-methylated internodes, including 4 writers (TRMT10C, TRMT61B, TRMT6, TRMT61A) and 2 erasers (ALKBH3 and ALKBH1) [22]. There were thirteen m5C regulators, including 11 writers [NSUN1 (NOP2), NSUN2, NSUN3, NSUN4, NSUN5, NSUN6, NSUN7, DNMT1, DNMT2 (TRDMT1), DNMT3A, DNMT3B], 1 reader (ALYREF), and 1 eraser (TET2) [23–26]. There are thirteen m6A RNA methylation regulators involved in the process of m6A transcript methylation (writers: METTL3, METTL14, WTAP, KIAA1429, RBM15, ZC3H13; readers: YTHDC1, YTHDC2, YTHDF1, YTHDF2, HNRNPC; erasers: FTO and ALKBH5) [27–32].

### Calculation of the m6A/m5C/m1A score

The m6A/m5C/m1A scores of samples were calculated based on the 32 m6A/m5C/m1A methylation regulators and gene set variation analysis (GSVA) [33, 34] analysis. The GSVA score for each of the 3 sets was only associated with the expression of the methylation modifiers and not the actual RNA methylation levels. Various m6A.The scores of m5C and m1A were used to rank the samples, and the median m6A-, m5C-, and m1A-scores of the samples were used to divide them into low- and high-score groups respectively. An analysis of Wilcoxon rank-sum tests was utilized to determine whether there was a significant association between each group and clinical indicators.

### Weighted gene co-expression network analysis

The lncRNAs associated with m6A, m5C and m1A scores were screened using the WGCNA package [35] in the TCGA database. All lncRNAs in the TCGA-glioma dataset were subjected to the goodSamplesGenes function to filter low-expression lncRNAs. A hierarchical cluster analysis of the TCGA glioma samples was performed using the WGCNA package, and no outliers were identified (Additional file 1: Fig. S1). Then, a hierarchical clustering tree was generated based on the selection of a soft threshold. For the identification of modules that were of interest, Pearson correlation coefficients were calculated between lncRNA modules and m6A/ m5C/ m1A score traits. The lncRNAs in the modules with the highest correlation coefficients were identified as m6A/m5C/m1Ascore-related lncRNAs.

### Differential expression analysis

To analyse differential expression, the R package edgeR (version 3.34.1) was used. First, overlapping lncRNAs in the TCGA and CGGA glioma datasets were selected. Then, overlapping lncRNAs that satisfied |log_2_-fold change (FC)|> 0.5 and P < 0.05 were identified by the R package edgeR between 3 normal and 672 glioma samples (glioma vs. normal) in TCGA, which were defined as DE-lncRNAs. To visualize the volcano plot of DE-lncRNAs, the R package ggplot2 was used (version 3.3.5).

### Construction, evaluation, and validation of the risk signature

M6A/m5C/m1A score-related lncRNAs and overlapping lncRNAs of DE-lncRNAs were considered candidate lncRNAs. We selected 667 glioma patients with complete survival information (nonzero survival time and survival status) from the TCGA-glioma dataset as a training set for screening prognostic lncRNAs and constructed the prognostic signature. The association of candidate lncRNAs with overall survival (OS) was evaluated by univariate Cox regression analysis. As a result, lncRNAs with P < 0.2 were considered significant variables associated with OS, and their results were then added into the multivariate Cox regression analysis to determine the optimal lncRNAs for the construction of the prognostic signature (multivariate Cox P < 0.2). By using multivariate Cox regression analysis, this study assessed the effectiveness of the prognostic signature by estimating the regression coefficient (coef) for selected prognostic lncRNAs. The formula for calculating the risk score is shown below.

$$risk\,score=h0\left(t\right)\times exp\left({\beta }_{1}{gene}_{1}+{\beta }_{2}{gene}_{2}+\dots +{\beta }_{n}{gene}_{n}\right),$$

where β refers to the regression coefficient and h0(t) is the baseline hazard function. The samples in the training cohort were divided into high- and low-risk groups based on a median risk score. For the CGGA cohort, we used the same formula to calculate and perform the classification of high- and low-risk groups. To demonstrate the predictive ability of different prognostic scoring models, they were tested separately on the training cohort and the validation dataset (CGGA cohort). Thereafter, the Kaplan‒Meier (K‒M) survival curves were used to assess the survival discrepancies between two groups. In addition, the receiver operating characteristic curve (ROC) was used to measure the accuracy of survival prediction at 1 year, 3 years, and 5 years using this signature.

### The independent prognostic value of the signature

Univariate and multivariate Cox regression analyses were performed to determine whether the prognostic signature was independently associated with clinical characteristics, in addition to other independent prognostic factors (P < 0.05). With the help of the R package rms (version 6.1-0), a nomogram was constructed based on these prognostic factors. Then calibration curve [36] was utilized to assess the effectiveness of the nomogram.

### Gene set enrichment analysis (GSEA) analysis

Gene Ontology (GO) term sets (c5.go.v7.4.entrez.gmt) and Kyoto Encyclopedia of Genes and Genomes (KEGG) pathway sets (c2.cp.kegg.v7.4.entrez.gmt) were downloaded from the GSEA database (http://www.gsea-msigdb.org/gsea/msigdb). As part of the analysis, the differentially enriched terms and pathways between two RNA methylation-related lncRNA risk groups were identified using the GSEA algorithm [37], which determines gene set differences between different phenotypic conditions based on genome-wide expression profiles.

### Immune landscape analysis

From published literature, we gathered the well-defined gene signatures of 24 immune cells [38]. Single-sample GSEA (ssGSEA) [39] was performed using the R package GSVA [34] to calculate the activity of immune cells or immune functions and immune pathways in each sample.

### Estimation of responses to immune checkpoint inhibitors (ICIs)

To predict the response to ICI therapy (anti-PD-1 and CTLA-4 therapies), the Tumour Immune Dysfunction and Exclusion (TIDE; http://tide.dfci.harvard.edu) algorithm and Wilcoxon test were used [40] to calculate and compared between high- and low-risk score groups, respectively. In addition, SubMap (https://cloud.genepattern.org/gp) algorithms were used to predict immune checkpoint response inhibitors of PD-1 and CTLA4 in low- and high-risk score groups.

### Chemotherapy response

The Genomics of Drug Sensitivity in Cancer (GDSC) database was used to predict the chemotherapy response of glioma patients. To evaluate the response of patients to chemotherapy drugs, the half-maximal inhibitory concentration (IC50) of the drug was calculated by the package pRRophetic.

### Gene Ontology and Kyoto Encyclopedia of genes and genomes enrichment analysis

To elucidate the potential gene functional annotation and pathway enrichment associated with the predicted miRNA-mRNA of two key lncRNAs. GO [41, 42] and KEGG [43] analyses were performed using the clusterProfiler (version 3.10.1) package [44]. The enrichplot and DOSE [45] packages were used to supply enrichment result visualization to help interpretation. P < 0.05 and adjusted P < 0.05 were set as the threshold values.

### Cell culture and transfection

Human glioma cells (LN229, U251, U87 and NHA) were purchased from the Chinese Academy of Sciences Cell Bank (Shanghai, China). Cell lines were cultured in Dulbecco’s modified Eagle’s medium (DMEM) or DMEM/F12 with 10% foetal bovine serum (Gibco, USA) in a humidified atmosphere of 5% CO_2_ at 37 °C. Human sh-RP11-98I9.4 and sh-RP11-752G15.8 plasmids were used to knockdown the lncRNA in the current study, whereas empty plasmid was used as a control. At 48 h after injection, G418 (800 μg/mL) was used to select stable LN229 and U251 cells. The plasmids were purchased from GeneChem (Shanghai, China). For knockdown of GBM cells, cells were transfected with plasmids using the Lipofectamine 2000 kit (Invitrogen, USA) according to the manufacturer’s instructions. The shRNA sequences used for RP11-98I9.4 and RP11-752G15.8 were as follows:

shRPRP11-98I9.4: 5′-ACCTTCGCCACAGAAACCAAG-3′

shRP11-752G15.8: 5′-GGAGCAAAGAGAGAAACAAGG-3′

shScramble: 5′-TTCTCCGAACGTGTCACGT-3′

### CCK-8 assay and drug treatment

GBM cell viability was evaluated with the Cell Counting Kit 8 (Sevenbio, Beijing, China) and was measured at OD 450 nm with the BioTek Gen5 system (BioTek, USA). For drug treatment, the cells were treated with various concentrations of TMZ for 72 h.

### Colony formation assay

A six-well plate was seeded with cells (0.3 × 10^3^ per well) and cultured for 11 days. After washing twice with PBS, the colonies were fixed with 4% formaldehyde for 10 min and stained with 0.1% crystal violet for 30 min. ImageJ was used to count the number of colonies captured with an Olympus camera (Tokyo, Japan).

### 5-Ethynyl-2′-deoxyuridine (EdU) assay

Cell proliferation was detected using an EdU Cell Proliferation Assay Kit (Beyotime, Shanghai, China) in accordance with the instructions provided by the manufacturer. An imaging microscope (Nikon C2, Tokyo, Japan) was used to determine the percentage of cells that incorporated EdU.

### RNA extraction, cDNA synthesis and quantitative real-time PCR

TRIzol reagent was used to isolate total RNA from cell lines (Invitrogen, 15596026.

In accordance with the manufacturer's protocol, cDNA was prepared using the PrimeScript RT Reagent Kit (TaKaRa; RR037). qRT‒PCR was performed in a Roche LightCycler 480 (Roche Diagnostics, USA) in quadruplicate and normalized to the glyceraldehyde 3-phosphate dehydrogenase (GAPDH) control. The primers used for qRT‒PCR were as follows:

RP11-752G15.8_forward: 5′-CCAAGAATTGCCAGACGCTT-3′

RP11-752G15.8_reverse: 5′-AGCTGCCCTTGTTTCTCTCT-3′

RP11-98I9.4_forward: 5′-GCAGCGCTCTGATTTACCAA-3′

RP11-98I9.4_reverse: 5′-GTTCCCGAACTAGAGTGGGT-3′

GAPDH_forward: 5′-GCACCGTCAAGGCTGAGAAC-3′

GAPDH_reverse: 5′-TGGTGAAGACGC CAGTGG A-3′

### Invasion assay

Cell invasion assays were conducted in 24-well cell culture chambers with Matrigel-coated Transwell inserts (Corning). A total of 1 × 10^4^ GBM cells were seeded per chamber. After 36 h, the lower surfaces of the chambers were fixed with 4% formaldehyde for 30 min and stained with 0.1% crystal violet.

### Dot blot analysis

By using a NanoDrop, the concentration of the purified RNA was measured and diluted to 100 ng/μl and 50 ng/μl with RNase-free water. We denatured purified RNA by heating it to 95 °C for 3 min and cooling it on ice. Nucleic acid transfer was optimized using the Hybond-N + membrane (Solarbio). Incubation with mouse anti-m5C antibody (1:1000; MAb-081-010, Diagenode) or rabbit anti-m6A antibody (1:1000, 202003, Synaptic Systems) was performed overnight at 4 °C after UV crosslinking. All membranes were incubated with HRP-labelled mouse IgG secondary antibodies (ZB-2305, Zsbio Store-bio) or rabbit IgG secondary antibodies (ZB-2316, Zsbio Store-bio) for 1 h before visualization by an imaging system (Bio-Rad, USA). An ECL Western blotting Detection Kit (Sevenbio, Beijing, China) was used to visualize the membrane. The other membrane was loaded with methylene blue.

### Xenograft model in vivo

Four-week-old female athymic BALB/c nude mice (n = 5 mice/group) were purchased from Cyagen (Suzhou, China). A total of 2.5 × 10^5^ GBM cells (LN229_Scramble, LN229_shRP11-98I9.4 and LN229_shRP11-752G15.8) per mouse were stereotactically injected into the brain. Fourteen days after GBM cell implantation, the mice were treated by oral gavage for 1 week with DMSO (0.3%) or TMZ (50 mg kg^−1^ day^−1^). The intracranial tumours were measured with bioluminescence imaging. All procedures were approved by Changshu No.1 People’s Hospital’s ethics committee.

### Statistical analysis

All statistical analyses were performed using GraphPad software version 7.0 (GraphPad Software), IBM SPSS Statistics 23.0 (SPSS) or software. Overlap analysis was performed in the Jvenn online analysis tool (http://jvenn.toulouse.inra.fr/app/example.html). Cox regression analysis was performed with the R package survival (version 3.2-7). K‒M survival curves were generated by the R package Survminer (version 0.4.8). The R package SurvivalROC (version 1.0.3) was utilized to produce ROC curves. All violin plots were plotted by the R package Vioplot (version 0.3.7). Key lncRNAs were identified by the LncBook 2.0 database. Statistical differences in nonnormally distributed variables were examined using the Wilcoxon test or Kruskal‒Wallis test. Student’s t test and one-way analysis of variance (one-way ANOVA) were used to assess differences between groups. P < 0.05 indicated statistical significance except unless otherwise specified.

## Results

### Evaluation of methylation scores in TCGA glioma patients

The m6A/m5C/m1A score of TCGA glioma was calculated by GSVA, and there were three main categories: m1A methylation score (containing 4 writers and 2 erasers); m5C methylation score (containing 11 writers, 1 reader, and 1 eraser); and m6A methylation score (containing 6 writers, 5 readers, and 2 erasers). The m1A methylation scores were distributed between -0.8624 and 0.8564; m5C methylation scores ranged from -0.7894 to 0.7412; and m6A methylation scores were located from -0.7685 to 0.7262 (Additional file 2: Table S1). A total of 672 glioma patients were classified into high- and low-score subgroups according to the cut-off points of each type of RNA methylation score (cut-off_m1A methylation score_ = − 0.0749, cut-off _m5C methylation score_ = − 0.02612, cut-off _m6A methylation score_ = − 0.03967, Additional file 1: Fig. S2). As shown in Additional file 1: Fig. S3, significant differences in m6A/m5C/m1A scores were detected in glioma patients with different clinical features. Specifically, m1A and m5C (Additional file 1: Fig. S3A) methylation scores were higher in patients with advanced age (age > 65), while m6A methylation scores (Additional file 1: Fig. S3A) increased in patients younger than or equal to 65 years. In addition, m6A methylation scores were significantly correlated with sex, with female patients having higher m6A methylation scores (Additional file 1: Fig. S3B), but m1A and m5C methylation scores were more evenly distributed between male and female samples. Consistently, nonsignificant associations (Additional file 1: Fig. S3C) were found between all three m6A/m5C/m1A scores and treatment modality (drug therapy or radiation therapy). In contrast, all three m6A/m5C/m1A scores were significantly correlated with the grade of glioma (Additional file 1: Fig. S3D).

### Identification of m6A/m5C/m1A score-related lncRNAs by WGCNA

**Fig. 1:**
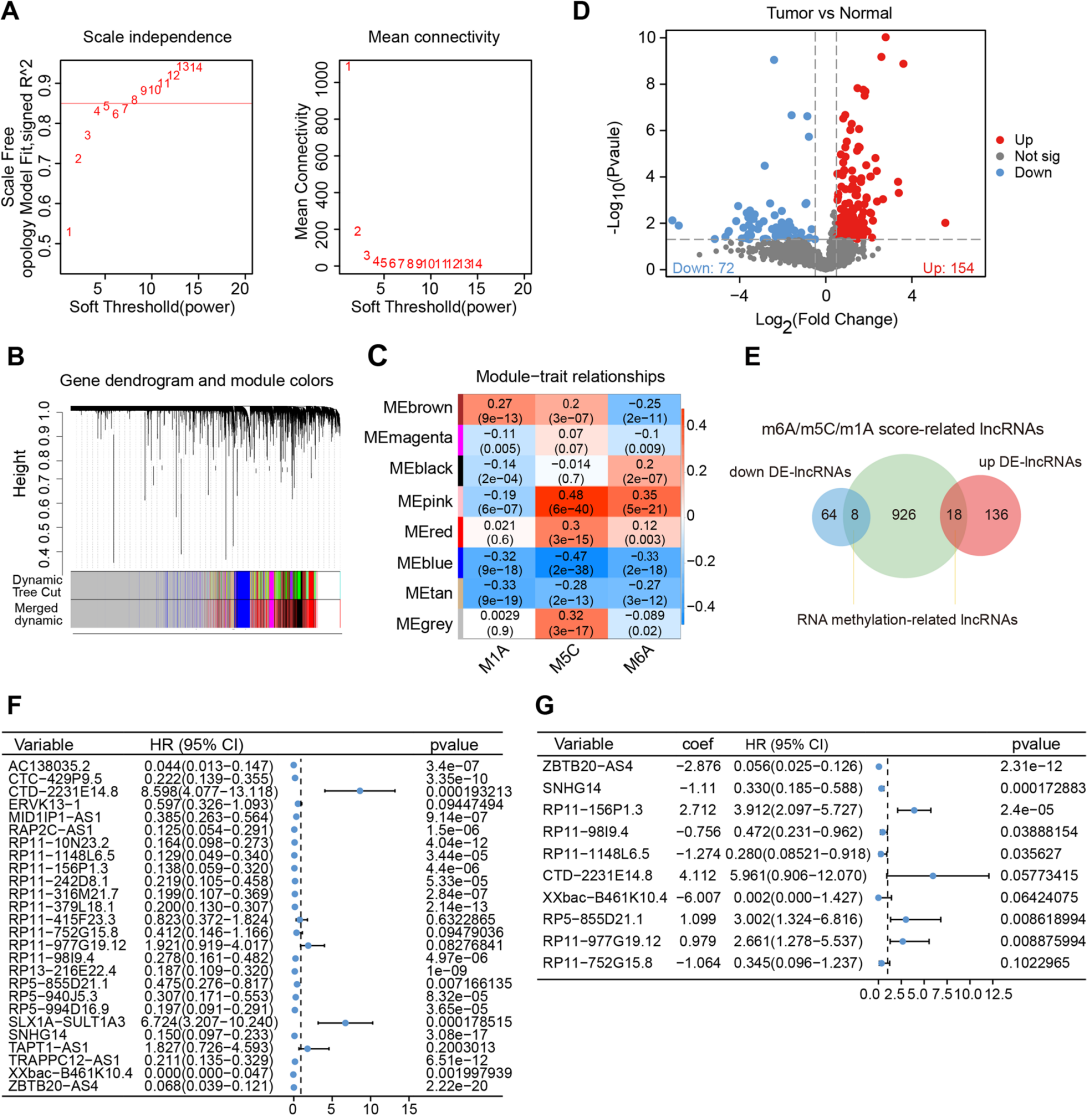
Construction of an RNA methylation-related lncRNA signature based on DE-m6A/m5C/m1A score-related lncRNAs associated with the survival of glioma patients. **A** Analysis of network topology for various soft-thresholding powers. **B** Gene dendrogram with different colours showing modules identified by WGCNA. **C** Relationship between gene modules and different m6A/m5C/m1A scores of gliomas. **D** Volcano plot of DE-lncRNAs between the normal and glioma groups in the TCGA database (selection criteria: P < 0.05 and |log_2_ FC|> 0.5). **E** Venn diagrams of RNA methylation-related lncRNAs by taking the intersections of significantly upregulated and downregulated DE-lncRNAs and m6A/m5C/m1A score-related lncRNAs. **F**, **G** The results of univariate Cox regression analysis (**F**) and multivariate Cox regression analysis (**G**) for the RNA methylation-related lncRNAs significantly associated with glioma OS. *DE-RNA* differentially expressed RNA, *WGCNA* weighted gene co-expression network analysis, *TCGA* The Cancer Genome Atlas, *OS *overall survival

Co-expression network analysis was performed on TCGA glioma lncRNA expression profiles (n = 672) using the R package WGCNA. In this study, we set a soft threshold of 8, at which point the scale-free R2 index reached 0.82 (Fig. 1A), ensuring a scale-free topology model criterion. Next, clustering analysis of TCGA-glioma was performed on this basis, where lncRNAs that exhibit similar expression patterns were grouped into identical modules, and a total of 7 modules were identified, while all non-co-expressed lncRNAs were gathered into grey modules (Fig. 1B). The results of Pearson correlation analysis between modules and different m6A/m5C/m1A scores of gliomas are shown in Fig. 1C, where the pink module was significantly correlated with m6A/m5C/m1A scores. Thus, 952 lncRNAs embedded in the pink module were selected based on their m6A/m5C/m1A scores (Additional file 3: Table S2).

### Identification of RNA methylation-related lncRNAs

First, we selected 3299 common lncRNAs from the TCGA and CGGA datasets. Next, DE-lncRNAs were screened in the TCGA database between the normal (n = 3) and glioma (n = 672) groups. A total of 154 genes were found to be upregulated and 72 genes were found to be downregulated (selection criteria: P < 0.05 and |log2 FC|> 0.5) between groups (Fig. 1D). A detailed list of genes is provided in Additional file 4: Table S3. Subsequently, we performed an overlap analysis of m6A/m5C/m1A score-related lncRNAs and DE-lncRNAs and obtained a total of 26 common lncRNAs (8 downregulated and 18 upregulated genes), which were termed RNA methylation-related lncRNAs (Fig. 1E; Additional file 5: Table S4).

### Construction of an RNA methylation-related lncRNA signature

Accordingly, to examine the relationship between the above 26 RNA methylation-related lncRNAs and the overall survival (OS) of glioma patients, we selected 667 glioma samples with complete survival data (nonzero survival time and documented survival status) from TCGA as the training cohort. The univariate Cox regression analysis revealed 24 lncRNAs that were significantly associated with glioma survival (all P < 0.2; Fig. 1F), all of which had hazard ratios (HRs) less than 1, indicating that they may be potential anti-oncogenes for glioma. Furthermore, multivariate Cox regression analysis based on the above 24 lncRNAs showed that ten lncRNAs (ZBTB20-AS4, SNHG14, RP11-156P1.3, RP11-98I9.4, RP11-1148L6.5, CTD-2231E14.8, XXbac-B461K10.4, RP5-855D21.1, RP11-977G19.12, and RP11-752G15.8) were the optimal variables for the construction of the prognostic signature (Fig. 1G).

### Assessment and validation of the validity of the ten lncRNA-based risk score

**Fig. 2:**
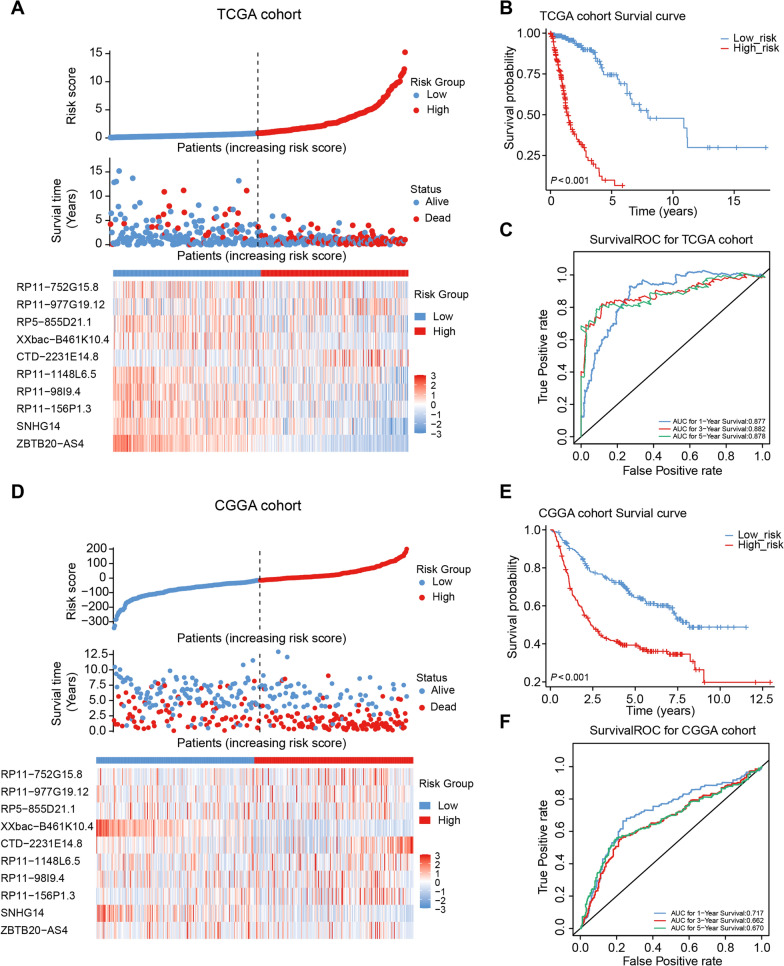
Evaluation and validation of the RNA methylation-related lncRNA signature. **A** RNA methylation-related lncRNA expression profiles, risk score distribution, and patients survival status in the TCGA cohort. **B** Kaplan‒Meier survival analysis between high-risk and low-risk groups in the TCGA cohort. **C** ROC curve evaluateing efficiency of the RNA methylation-related lncRNA signature for predicting 1-, 3-, and 5-year OS in the TCGA cohort. **D** RNA methylation-related lncRNA expression profiles, risk scores distribution, and patients survival status in CGGA cohort. **E** Kaplan‒Meier survival analysis between two groups in the CCGA cohort. **F** ROC curves of 1/3/5-year in the CGGA cohort. CGGA, Chinese Glioma Genome Atlas; ROC, receiver operating characteristic; AUC, area under the curve

Based on the expression of selected prognostic lncRNAs and their regression coefficients (coef), a risk scoring system was constructed. In the training cohort and validation cohort (CCGA-glioma dataset; n = 313), the median risk score was calculated for glioma samples and set as the cut-off value for risk stratification of glioma patients (Fig. 2A, D). K‒M curves were used to evaluate whether there was a significant difference between patients with high-risk and low-risk gliomas in terms of survival time. The results demonstrate that high-risk scores were notably linked to poor clinical outcomes (all P < 0.001; Fig. 2B, E). In the training cohort, further evidence implied that the areas under the ROC curves for evaluating 1-year, 3-year, and 5-year OS were 0.877, 0.882, and 0.878, respectively (Fig. 2C). In the validation cohort, the AUC values were 0.717, 0.662, and 0.670 for glioma patients at 1, 3, and 5 years, respectively (Fig. 2F). The above evidence suggested that the constructed RNA methylation-related lncRNA prognostic signature based on these ten lncRNAs had tolerable prognostic predictive validity and acceptable applicability. Moreover, according to the 2016 and 2021 WHO classification of CNS tumours [46, 47], gliomas have been classified as IDH1 wild-type (GBM), IDH1 mutations with chromosome 1p19q codeletions (LGG), and IDH1 mutations without chromosome 1p19q codeletions (LGG). We validated our model separately in two groups of patients, the results also demonstrated that high-risk scores were notably linked to poor clinical outcomes (Additional file 1: Fig. S4A). In addition, the relationship of risk score and several well-known biomarkers of glioma (IDH mutant, 1p19q co-deleted, and methylation of MGMT promoter) was analyzed to verify the clinical applicability of the signature. Additional file 1: Fig. S4B demonstrated that risk score was significantly lower in the status of IDH mutant, 1p19q co-deleted gliomas, and MGMT promoter methylation (p < 0.05).

### Risk score and age were independent prognostic factors for glioma patients

**Fig. 3:**
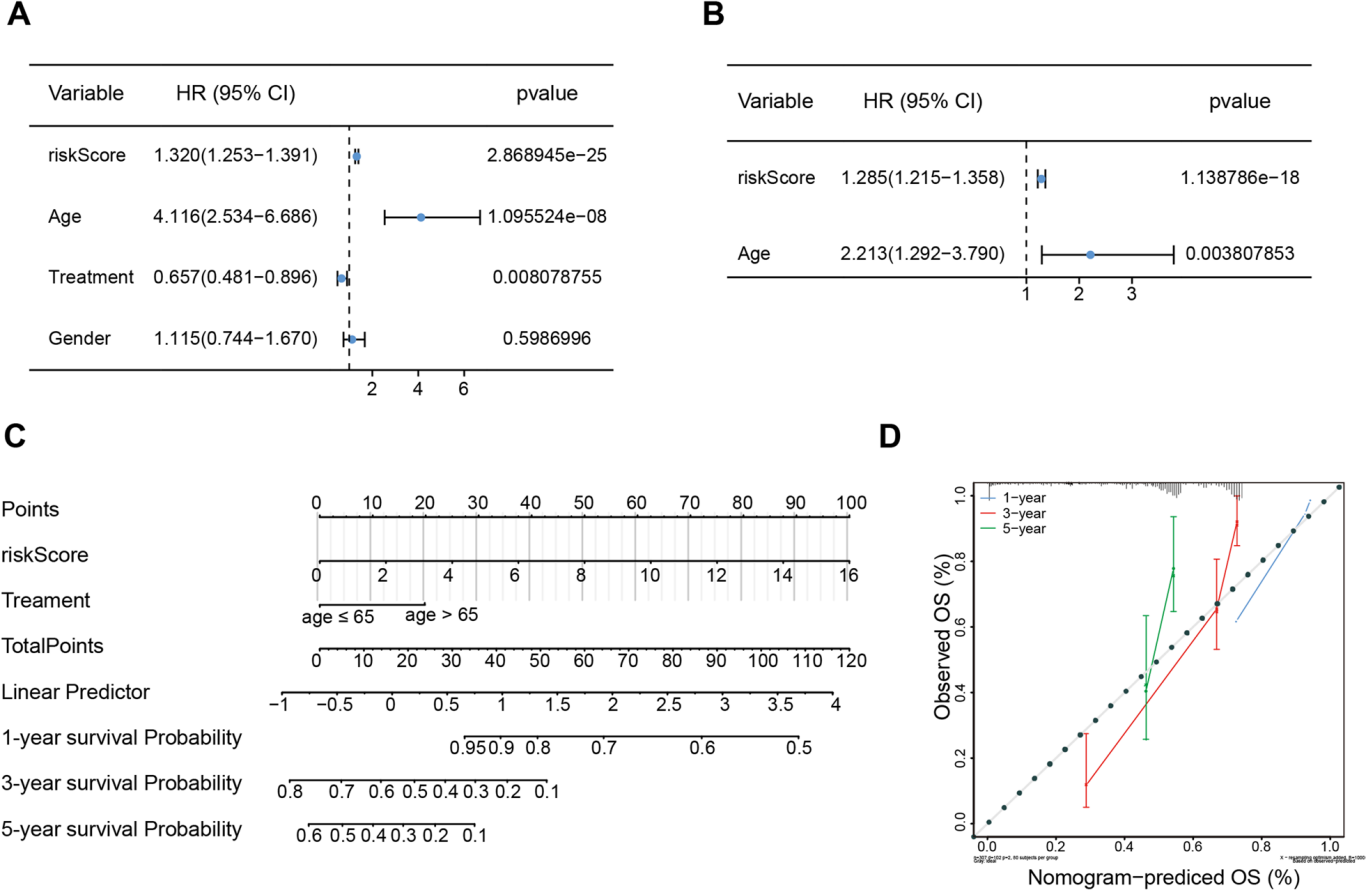
Independent prognostic analysis of the RNA methylation-related lncRNA signature in glioma. **A**, **B** Univariate (**A**) and multivariate (**B**) Cox regression analyses to identify independent prognostic factors among RNA methylation-related lncRNAs and other clinicopathological characteristics in the TCGA database (P < 0.05). **C** Nomogram for 1-, 3-, and 5-year overall survival prediction of glioma patients. **D** The calibration curves of the nomogram

We first evaluated the significance of different clinicopathological characteristics of patients according to the risk score in TCGA and analysed the association between the risk score and the clinical characteristics (Table 1). It was found that the risk scores varied significantly among different ages and tumour classifications. Furthermore, to explore whether our risk score could perform independently of other clinical characteristics, such as age, sex, and treatment type, we performed a Cox regression analysis on the TCGA-glioma dataset. Univariate Cox regression analysis revealed that risk score, age, and treatment type had prognostic value in the TCGA-glioma dataset (all P < 0.05; Fig. 3A). We then included the above three indicators in a multivariate Cox regression analysis. As a result, we determined that the risk score and age had independent prognostic significance (all P < 0.05; Fig. 3B). In accordance with these results, we developed a nomogram integrating the identified independent prognostic factors (risk score and age), which could be utilized to predict the 1-, 3-, and 5-year survival rates of gliomas (Fig. 3C). The C index of the nomogram was 0.8281. Simultaneously, the calibration curve of this nomogram was developed and is presented in Fig. 3D.

**Table 1:** The clinical characteristics of the two risk groups in TCGA

Characteristics	High_risk	Low_risk	P value
Total number, N	206	206	
Gender, n (%)			0.9205
Male	120 (29.1%)	119 (28.9%)	
Female	86 (20.9%)	87 (21.1%)	
Age, n (%)			< 0.001
Age > 65	43 (10.4%)	12 (2.9%)	
Age ≤ 65	163 (39.6%)	194 (47.1%)	
Treatment, n (%)			0.6221
Pharmaceutical therapy	98 (23.8%)	103 (25%)	
Radiation therapy	108 (26.2%)	103 (25%)	
Project, n (%)			< 0.001
TCGA-GBM	142 (34.5%)	78 (18.9%)	
TCGA-LGG	64 (15.5%)	128 (31.1%)

### Recognition of key lncRNAs

We first identified 10 unique codes for prognostic lncRNAs in the Ensembl database (https://grch37.ensembl.org) and then predicted the interactions of lncRNAs with microRNAs (miRNAs) and RNA Binding Protein (RBP) based on the LncBook 2.0 database. Among them, RP11-98I9.4 and RP11-752G15.8 were predicted to be linked to miRNAs in the LncBook database and interacted with the malignant cell line RBP and were therefore defined as key lncRNAs. Based on the 2 key prognostic lncRNAs, the miRNAs predicted in the miRanda, TargetScan and RNAhybrid databases were selected as the targeting miRNAs of key lncRNAs, and RBP was selected as the default value to obtain the regulatory network of key lncRNAs with miRNAs and RBPs (Additional file 1: Fig. S5A). To further investigate the clinical implications of two key lncRNAs, we compared the survival rate and pathological grading between the high- and low-expression groups of them. The K‒M curves revealed that the survival rates were significantly different between the high- and low-expression groups of RP11-98I9.4 and RP11-752G15.8 (p < 0.05), and the low expression groups were accompanied by a poorer prognosis (Additional file 1: Fig. S5B). In addition, Additional file 1: Fig. S5C demonstrated that the expression of two key lncRNAs was lower in GBM than in LGG (p < 0.05). Thus, above results indicated that RP11-98I9.4 and RP11-752G15.8 were correlated with the prognosis of glioma patients.

### Molecular mechanisms underlying the risk score in glioma progression

**Fig. 4:**
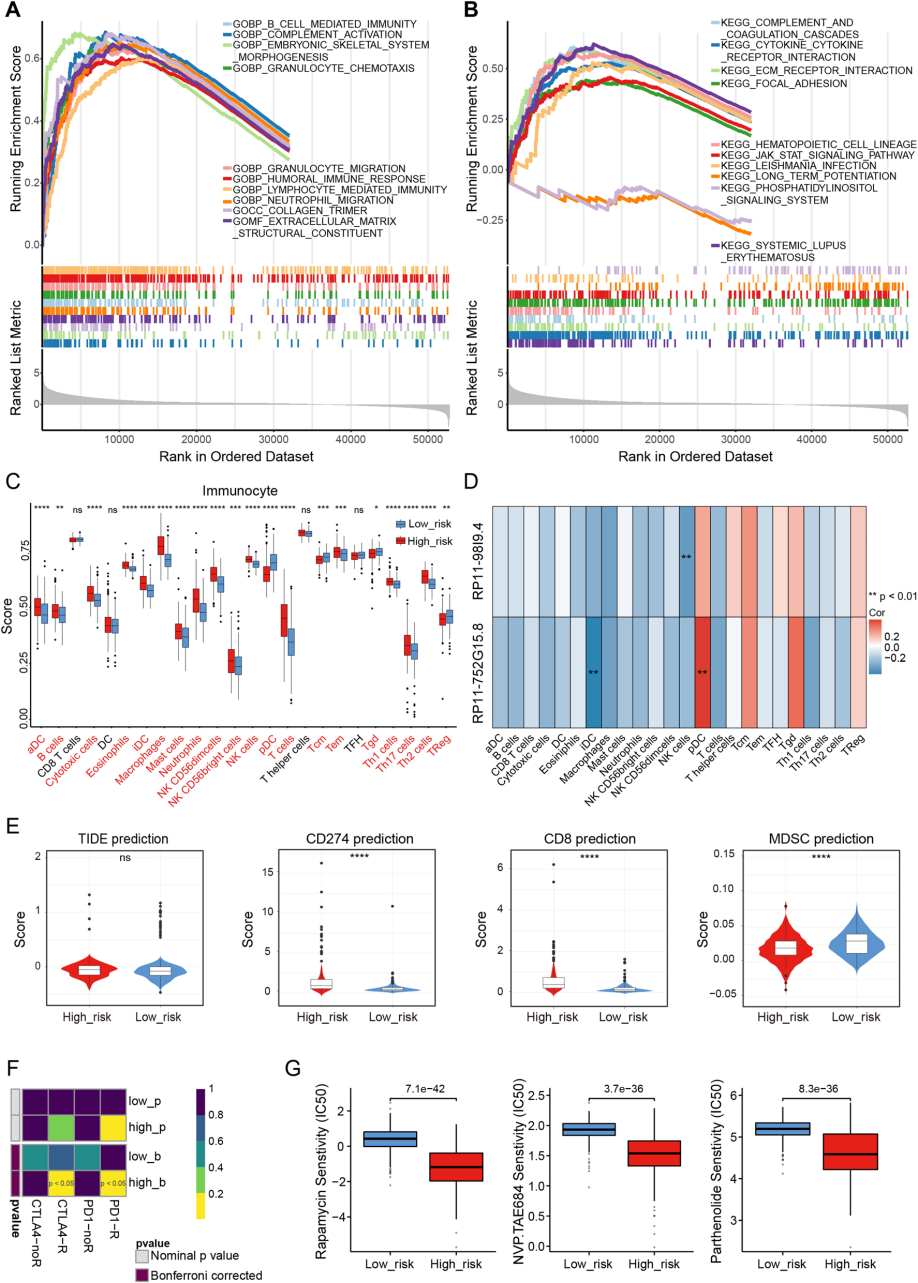
Comprehensive analysis of the RNA methylation risk score in glioma. GSEA between the low- and high-risk groups, including the top 10 GO terms (**A**) and KEGG pathways (**B**). **C** Differences in the abundance of 24 immune cells between the low- and high-risk groups (Kruskal‒Wallis test). **D** Pearson correlation analysis between RNA methylation-related lncRNAs and 24 immune cells in the TCGA database. **E** Differences in responses to ICI therapy (anti-PD-1 and CTLA-4 therapies) between the low- and high-risk groups using the TIDE algorithm. *P < 0.05; **P < 0.01; ***P < 0.001; ****P < 0.0001. **F** Heatmap of ICI expression for PD-1 and CTLA4 in the low- and high-risk score groups through SubMap. **G** Differences in the IC50 values of three chemotherapeutic drugs for glioma between the low- and high-risk groups. *GSEA* Gene Set Enrichment Analysis, *GO* Gene Ontology, *KEGG* Kyoto Encyclopedia of Genes and Genomes, *ICI* immune checkpoint inhibitor, *IC50* half-maximal inhibitory concentration

To better understand the biological functions of the risk score, GSEA was applied to analyse the possible functions and pathways of all genes between the high- and low-risk groups. According to |NES|> 1, P < 0.05, and q < 0.25, the GO analysis enriched a total of 697 terms (Additional file 6: Table S5). As shown in Fig. 4A, 5of the top 10 GO terms associated with immune and inflammatory responses were significantly enriched in the high-risk group (COMPLEMENT_ACTIVATION, B_CELL_MEDIATED_IMMUNITY, GRANULOCYTE_CHEMOTAXIS, HUMORAL_IMMUNE_RESPONSE, and LYMPHOCYTE_MEDIATED_IMMUNITY). KEGG analysis revealed a total of 13 pathways significantly associated with the risk score (Additional file 7: Table S6). The top 10 KEGG pathways are shown in Fig. 4B and were significantly relevant to the immune and inflammatory response (‘COMPLEMENT AND COAGULATION CASCADES’, ‘CYTOKINE CYTOKINE RECEPTOR INTERACTION’, ‘JAK STAT SIGNALING PATHWAY’, etc.). Moreover, the risk score may also be involved in the cell cycle (‘CELL CYCLE’). These evidence suggests that the risk score might affect OS in glioma patients by modulating immune/inflammatory responses and glial cell physiological processes.

### Relationship between risk score and immune infiltrating cells

Inspired by the above results, we assessed the abundance of 24 immune cells in TCGA glioma by ssGSEA. The Kruskal‒Wallis test showed that the abundances of pDCs, Tcm cells, Tgd cells, and TReg cells were more pronounced in the low-risk group, whereas the remaining immune cells showed a positive correlation with the risk score and a high level of infiltration in the high-risk group **(**Fig. 4C**)**. Additionally, Pearson correlation analysis (Fig. 4D; Additional file 8: Table S7) revealed that lncRNA RP11-752G15.8 had a weak positive correlation with pDCs (cor = 0.36483, P = 2.13E−23) and a weak negative correlation with iDCs (cor = − 0.39772, P = 7.14E−28). Furthermore, lncRNA RP11-98I9.4 had a weak negative correlation with NK cells (cor = − 0.3129, P = 2.56E−17). According to these results, the RNA methylation-related lncRNA signatures were associated with increased immune infiltration in glioma patients at high risk.

### Prediction of immunotherapy responsiveness and targeted drug sensitivity

With the help of the TIDE algorithm, we also assessed the predictive capability of the RNA methylation-related lncRNA signature in predicting the immunotherapy. Patients with low risk scores had lower CD274 and CD8 prediction scores and higher MDSC prediction scores (P < 0.0001; Fig. 4E). It should be noted that when comparing the expression profiles of the low- and high-risk groups with the expression profiles of glioma patients who had responded to immunotherapies in SubMap modules, the high-risk group showed a potential response to anti-PD-1 and anti-CTLA4 therapy (Bonferroni-corrected P < 0.05; Fig. 4F). Furthermore, we attempted to determine whether the 10-lncRNA prognostic signature was applicable not only to immunotherapies but also to chemotherapies to improve their predictability. There was a significant difference in chemotherapeutic drug sensitivity between the high-risk and low-risk groups for 100 of the 123 anticancer drugs. Rapamycin (P = 7.1e−42), NVP. TAE684 (P = 3.7e−36) and parthenolide (P = 8.3e−36) exhibited potential therapeutic value for glioma (Fig. 4G). The differences in IC50 between the two risk groups for the remaining 97 small molecule compounds/drugs are shown in Additional file 1: Fig. S6.

### Strong associations between the relative expression of the two RNA methylation-related lncRNAs and glioma

**Fig. 5:**
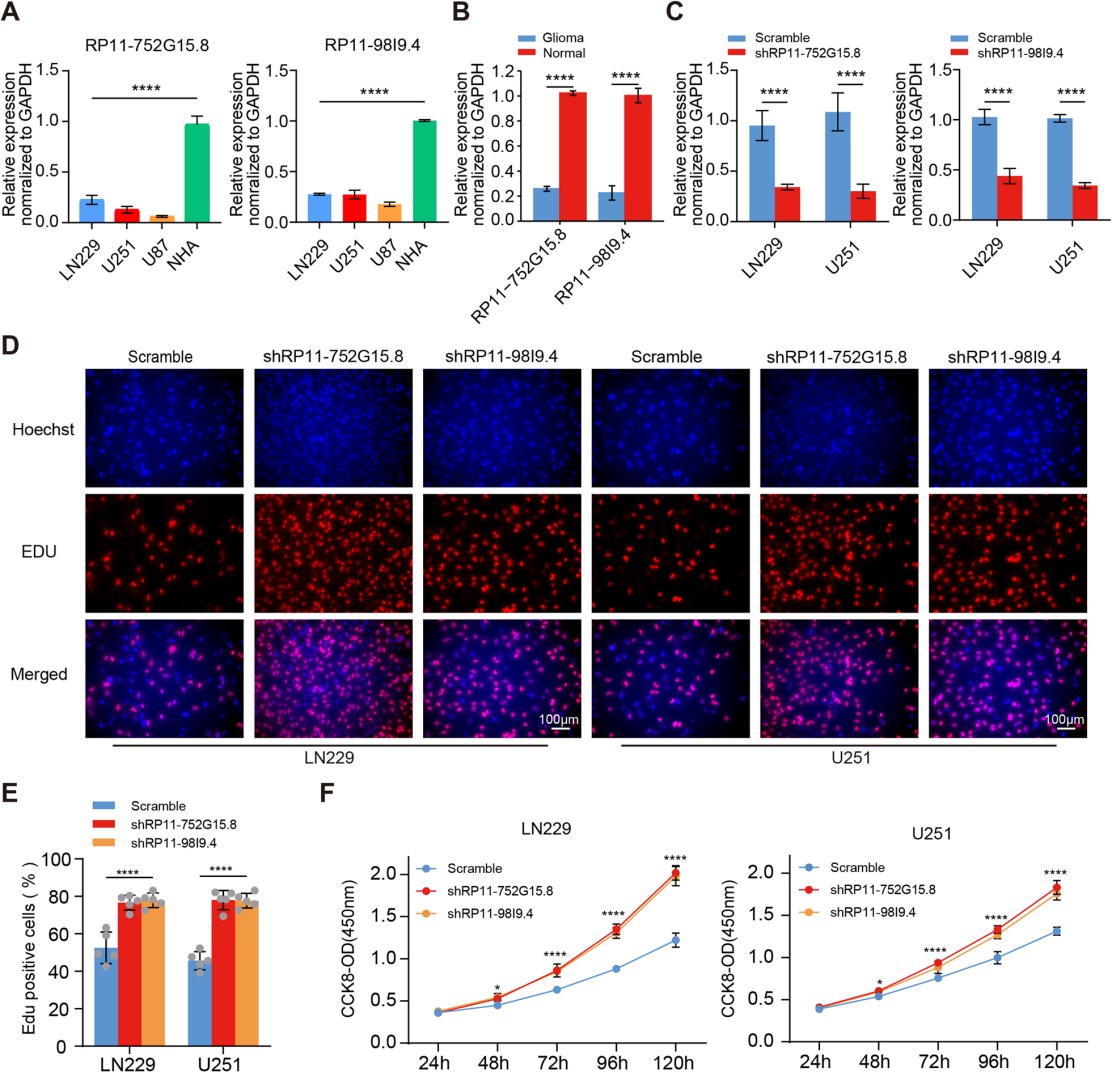
Knockdown of RP11-98I9.4 and RP11-752G15.8 promotes proliferation. **A** qRT‒PCR was used to detect the expression of RP11-98I9.4 and RP11-752G15.8 in LN229, U251, U87 and hmc3 cells. **B** qRT‒PCR was used to detect the expression of RP11-98I9.4 and RP11-752G15.8 in glioma tissues and tumor-adjacent tissues. **C** qRT‒PCR was used to detect the efficiency of shRP11-98I9.4 and shRP11-752G15.8 in LN229 and U251 cells. **D**–**F** EdU and CCK8 assays were used to assess the proliferative capacities of LN229 and U251 cells after RP11-98I9.4 and RP11-752G15.8 knockdown. Scale bar = 100 μm. *P < 0.05; **P < 0.01; ***P < 0.001; ****P < 0.0001

**Fig. 6:**
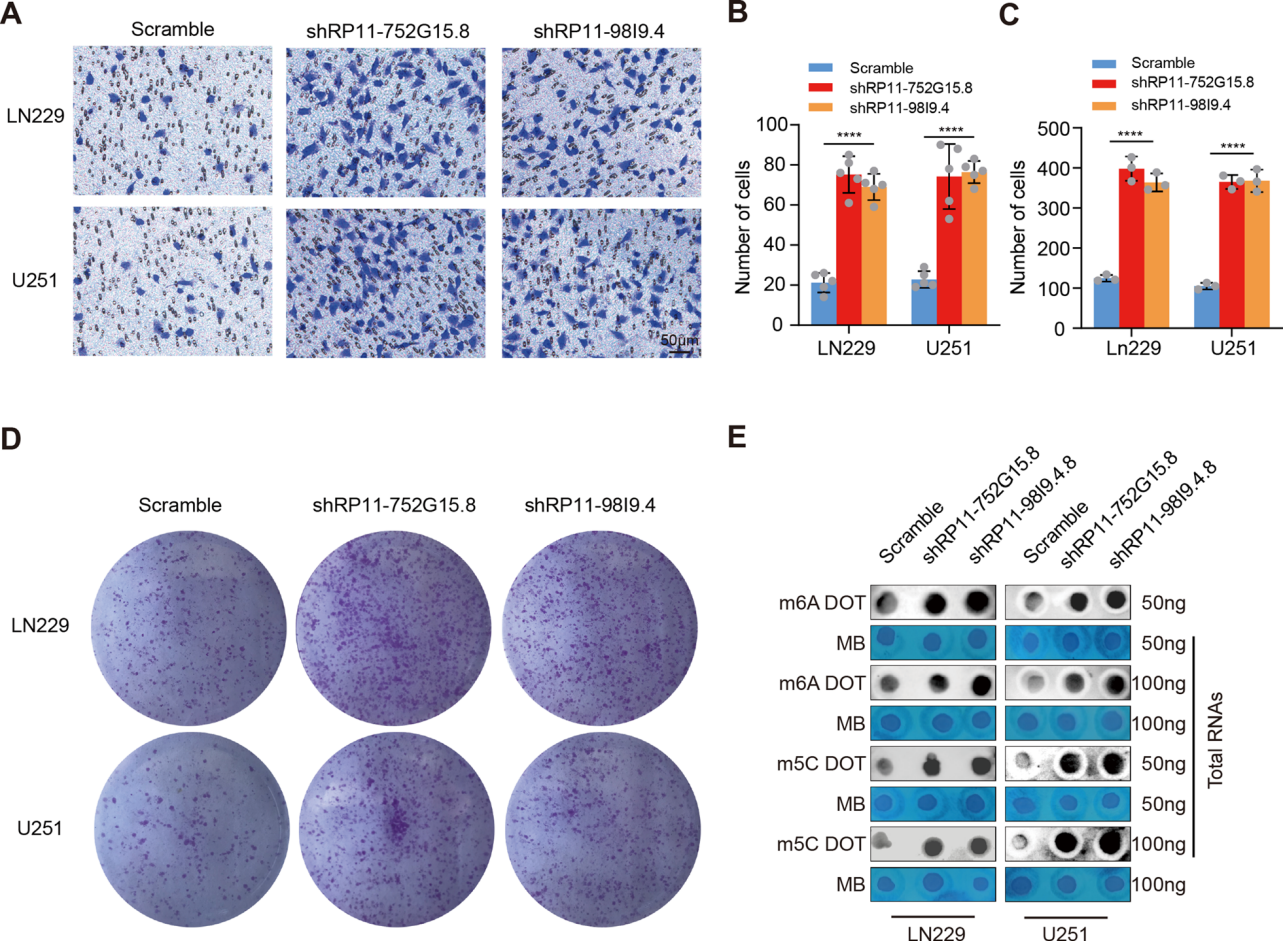
Knockdown of RP11-98I9.4 and RP11-752G15.8 enhance invasion and colony formation and increases global RNA m5C and m6A levels in glioma cells. **A**, **B** Transwell assays were used to assess the invasion capacities of LN229 and U251 cells after RP11-98I9.4 and RP11-752G15.8 knockdown. Scale bar = 50 μm. **C**, **D** Colony formation assay was used to assess the colony formation capacities of LN229 and U251 cells after RP11-98I9.4 and RP11-752G15.8 knockdown. **E** Representative dot blot images showing m5C and m6A abundance after RP11-98I9.4 and RP11-752G15.8 knockdown. *P < 0.05; **P < 0.01; ***P < 0.001; ****P < 0.0001

To investigate whether RP11-98I9.4 and RP11-752G15.8 play a role in glioma, three human glioma cells (LN229, U251, and U87), one human astrocyte cell line (NHA) were subjected to RT-PCR. Strikingly, quantitative analysis revealed that the expression of RP11-98I9.4 and RP11-752G15.8 was significantly lower in the glioma cell lines in comparison to NHA cell line (Fig. 5A). In addition, we detected the expressions of the two key lncRNAs in glioma samples of four pairs of glioma tissues and tumours-adjacent tissues collected in clinic. We further confirmed the expression of RP11-98I9.4 and RP11-752G15.8 had lower levels in glioma specimens than those in tumour-adjacent tissues, as consistent with the result of glioma cell lines (Fig. 5B). To determine whether RP11-98I9.4 and RP11-752G15.8 also affect the malignant behaviours of glioma, we constructed LN229 cells and U251 cells that had downregulated RP11-98I9.4 and RP11-752G15.8 expression. RT‒PCR results showed that interference of RP11-98I9.4 and RP11-752G15.8 had acceptable efficiency (Fig. 5C). Knockdown of RP11-98I9.4 and RP11-752G15.8 promoted proliferation (Fig. 5D–F) and enhanced invasion colony formation (Fig. 6A–D). Next, we examined whether interference of RP11-98I9.4 and RP11-752G15.8 affected m5C and m6A levels. Dot blot assays revealed that global RNA m5C and m6A levels were significantly elevated after RP11-98I9.4 and RP11-752G15.8 were downregulated in glioma cells (Fig. 6E).

**Fig. 7:**
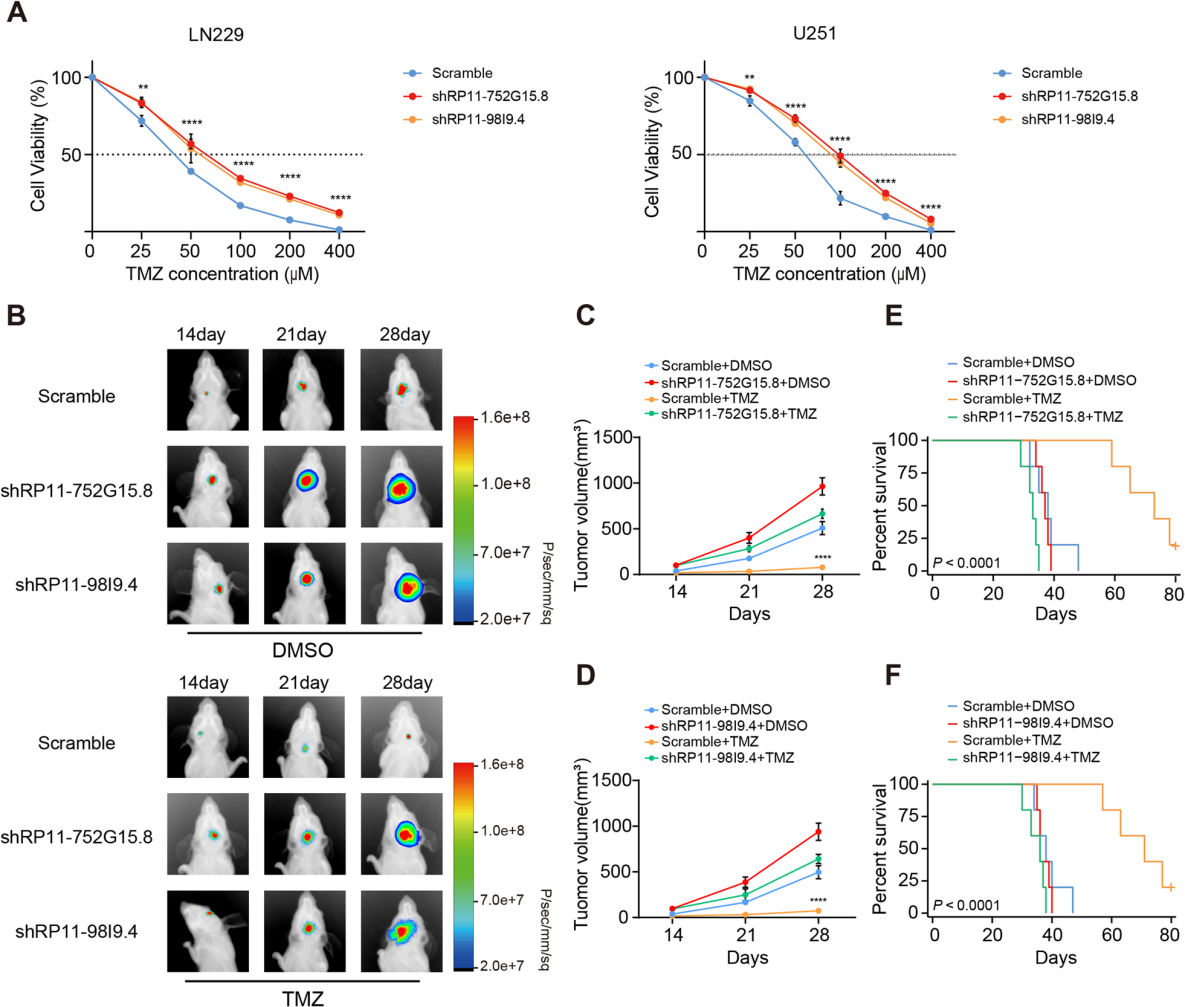
Knockdown of RP11-98I9.4 and RP11-752G15.8 promotes TMZ resistance in glioma cells. **A** CCK-8 assay was used to reveal the effect of RP11-98I9.4 and RP11-752G15.8 knockdown on glioma cells treated with TMZ at the indicated concentrations for 72 h. **B** Representative bioluminescence images of intracranial xenografts bearing LN229_shRP11-98I9.4, LN229_shRP11-752G15.8 or LN229_Scramble cells in DMSO or the presence of TMZ treatment on the indicated days. **C**, **D** Tumour volume was detected in each mouse group. **E**, **F** Kaplan‒Meier survival curve of each group is shown. *P < 0.05; **P < 0.01; ***P < 0.001; ****P < 0.0001

To investigate the functional role of RP11-98I9.4 and RP11-752G15.8 in TMZ resistance. There was a significant increase in the IC50 of the antitumour drug TMZ when RP11-98I9.4 and RP11-752G15.8 were knocked down in glioma cells from U251 and LN229, which counteracted the inhibitory effect of TMZ on tumour cell growth (Fig. 7A). To evaluate the effect of RP11-98I9.4 and RP11-752G15.8 on the TMZ-resistant phenotype in vivo, 2.5 × 10^5^ luciferase-labelled LN229_shRP11-98I9.4 or LN229_shRP11-752G15.8 and LN229_Scramble cells were injected into nude mice. Fourteen days after glioma cell implantation, the mice were treated by oral gavage for 1 week with DMSO (0.3%) or TMZ (50 mg kg^−1^ day^−1^). In vivo bioluminescence imaging was used to track the proliferation of tumours. As expected, xenografts bearing LN229_shRP11-98I9.4 or LN229_shRP11-752G15.8 cells displayed significant tumour growth promotion even with TMZ treatment (Fig. 7B–D). Although TMZ treatment reduced tumour burden, tumour size in the shRP11-98I9.4 and shRP11-752G15.8 TMZ group was still relatively increased compared to that in the LN229_Scramble DMSO group (Fig. 7B–D). Mice with shRP11-98I9.4 or shRP11-752G15.8 cells exhibited significantly poorer survival (Fig. 7E, F). Taken together, these findings indicate that knockdown of RP11-98I9.4 and RP11-752G15.8 induces a more invasive phenotype, accelerates cell growth and apparent resistance to TMZ both in vitro and in vivo, and improves global RNA m6A and m5C levels in glioma cells.

### GO and KEGG analysis of two lncRNAs predicted regulatory genes

To explore what happened in the downstream genes after RP11-98I9.4 and RP11-752G15.8 were knocked down. With the assistance of the miRNA predicted by the previous analysis, we used the miRTarBase database (http://mirtarbase.mbc.nctu.edu.tw/index.html) to further predict miRNA-mRNA (Additional file 9: Table S8) for each key lncRNA, and then performed enrichment analysis on mRNAs. The results of GO terms and KEGG pathways enrichment analysis are in Additional file 1: Fig. S7 and Additional file 10: Table S9. GO and KEGG pathway analyses identified that significantly enriched pathways in predicted miRNA-mRNA of two key lncRNAs, including negative regulation of single stranded viral RNA replication via double stranded DNA intermediate, ribonucleoside metabolic process, ribonucleoside metabolic process, toll-like receptor 9 signaling pathway, GnRH secretion, Sphingolipid signaling pathway, MAPK signaling pathway and so on in RP11-752G15.8 (Additional file 1: Fig. S7A, B). Similarly, we found that EMC complex, membrane insertase activity, glycerolipid metabolic process, regulation of nuclear-transcribed mRNA poly(A) tail shortening, glycerophospholipid metabolic process, phospholipid metabolic process, Central carbon metabolism in cancer, HIF-1 signaling pathway, Hippo signaling pathway—multiple species and so on in RP11-98I9.4 (Additional file 1: Fig. S7C, D).

## Discussion

Both mRNAs and lncRNAs undergo methylation modifications that are among the most common epigenetic modifications [48]. In addition, a number of lncRNAs have been shown to directly or indirectly regulate some methylation modification processes [49, 50]. Among epigenetics research hotspots, methylation modification is at the forefront. Methylation modification has been shown to be closely associated with the prognosis, immune regulation, and drug sensitivity of various tumour types through numerous clinical and preclinical trials [51]. A wide range of cancer-associated processes are associated with lncRNAs, including those associated with miRNA silencing, epigenetic regulation, DNA damage, cell cycle regulation, and signal transduction pathways [52]. Additionally, a growing amount of research suggests that lncRNAs play a crucial role in the regulation of tumorigenesis, proliferation, aggression, metastasis, and drug resistance in gliomas [53]. lncRNAs and the methylation of RNA may interact in tumours in the current state of knowledge. In the progression and malignancy of hepatocellular carcinoma, NSUN2-mediated m5C modification of H19 lncRNA plays an important role in the process of mutation of the lncRNA [54]. Cancer cells cannot proliferate, migrate, or invade in vitro or in vivo if their lncRNA, THOR, is lost, while the m6A readers YTHDF1 and YTHDF2 can regulate THOR, thus inhibiting tumour formation in vivo and in vitro [55]. CBS mRNA stability is decreased in a m6A-dependent manner by HIF-1a, inducing lncRNA-CBSLR to recruit the YTHDF2 protein and CBS mRNA into CBSLR/YTHDF2/CBS complexes. Because CBS expression was reduced, the methylation of the ACSL4 protein was reduced, and the protein was degraded via ubiquitination-proteasomes. In a hypoxic tumour microenvironment, it eventually protects gastric cancer cells from ferroptosis [56]. Knockdown of lncRNA NUTM2A-AS1 suppresses lung adenocarcinoma cell viability and induces apoptosis through the miR-590-5p/METTL3 axis [57]. Overall, numerous experimental findings indicate that lncRNAs regulated by RNA methylation play a critical role in bringing about, developing, and metastasizing cancerous tumours. However, it is still unclear how RNA methylation-related lncRNAs are involved in gliomas.

In the existing research, a number of studies have been reported to be suboptimal on RNA methylation-related lncRNA prognostic models. Xie et al. [58] developed a model that predicted the prognosis of patients with GBM by using m6A-associated lncRNAs. In addition to prognostic prediction, Liu et al. [59] also used lncRNAs implicated in m5C to analyse immune response signatures for disease progression. Shao et al. [60] demonstrated various aspects of glioma prognosis, the immune microenvironment, the tumour microenvironment (TME), and drug sensitivity related to lncRNAs associated with m6A/m5C/m1A/m7G. We hope to improve these prognostic signatures for glioma. In this study, using TCGA data of glioma, a predictive risk signature of RNA methylation-related lncRNAs was constructed through differential expression analysis, as well as WGCNA, resulting in greater validity and accuracy of the signature, which was validated by multiple verifications, demonstrating its validity and applicability. In detail, we integrated the data of m6A/m5C/m1A-related genes to calculate m6A/m5C/m1A scores by GSVA. Based on the m6A/m5C/m1A score, we first used differential expression analysis and WGCNA to identify associations with differentially expressed RNA methylation-related lncRNAs. Ten RNA methylation-related lncRNAs were obtained: ZBTB20-AS4, SNHG14, RP11-156P1.3, RP11-98I9.4, RP11-1148L6.5, CTD-2231E14.8, XXbac-B461K10.4, RP5-855D21.1, RP11-977G19.12, and RP11-752G15.8. Subsequently, ten RNA methylation-related lncRNAs associated with OS were screened and used to develop a risk score signature by univariate and multivariate Cox regression analysis. The ROC curves, K‒M curves, and nomogram were drawn to verify the accuracy of this signal model in more detail. It was found that this model was capable of predicting the risk score of the data with an effective and accurate level of accuracy. In addition to the CGGA cohort, survival analysis and ROC curves were validated. Furthermore, a prognostic signature comprising relevant lncRNAs was constructed based on methylation scores, and its prognostic value and relationship with immune function, immune therapy and drug sensitivity were determined. GSEA was applied to analyse the possible functions and pathways of all genes between the high- and low-risk groups. Finally, we demonstrated the feasibility of using the prognostic features of glioma patients through in vitro and in vivo experiments. As we progress in our research, we hope to clarify their prognostic value and explain how they contribute to the carcinogenesis and development of gliomas.

Among the ten lncRNAs we obtained, only SNHG14 had been previously studied. SNHG14/miR-5590-3p/ZEB act as a positive feedback loop in diffuse large B-cell lymphoma (DLBCL) cells to activate PD-L1, thereby inactivating CD8 + T cells and enhancing immune evasion. DLBCL cells were stimulated by SNHG14 in vitro and in vivo to proliferate, invade, undergo epithelial-mesenchymal transition (EMT) and grow [61]. OGD/R-induced neuronal injury in HT22 mouse hippocampal neuronal cells is exacerbated by the lncRNA SNHG14, which induces excessive mitophagy through miR-182-5p/BINP3 signalling [62]. However, the interaction of SNHG14 with methylation has not been studied. In our study, we demonstrated that RP11-98I9.4 and RP11-752G15.8 suppress glioma tumours. Glioma cells exhibited increased proliferation rates, enhanced migration, higher RNA methylation levels and more obvious resistance to TMZ when RP11-98I9.4 and RP11-752G15.8 were knocked down. There has been no clarification regarding the significance of the remaining lncRNAs as of yet, and this provides directions for future research and providing a framework for which the remaining lncRNAs can be studied. We intend to further research these genes in the future to determine if they have any relationship with RNA methylation as well as to further evaluate their significance in relation to glioma invasion, migration, proliferation and TMZ resistance in the future.

Nevertheless, there are still some limitations to our study that need to be addressed. For instance, we used TCGA and CGGA databases to obtain our glioma data. There is a need for further validation of the model in additional glioma cohorts. In addition, the predictive value of RNA methylation-related lncRNAs for clinical applications requires additional evaluation. It is necessary to investigate in more depth what other lncRNAs function and how they interact in gliomas.

## Conclusion

Gliomas have a poor prognosis, and improving the prognosis is of particular importance in this regard. m6A/m5C/m1A RNA methylation has been demonstrated to be involved in the progression of cancer in studies. To develop new targets for the diagnosis and treatment of cancer, many studies are using lncRNAs to establish prognostic markers. In this study, we demonstrate the correlation between lncRNAs associated with m6A/m5C/m1A and glioma prognosis, immune infiltrating cells, immunotherapy responsiveness, and drug sensitivity to targeted therapy. It is possible to use the prognostic signature as an independent factor for improving the prediction of glioma prognoses, which may potentially be an option for immunotherapy in the future.

### Supplementary Information

**Additional file 1: Figure S1**. Hierarchical clustering of the TCGA glioma samples. **Figure S2**. The TCGA cohort was classified into high- and low-scoring subgroups according to the m6A/m5C/m1A score of each type. **Figure S3**. Differences in clinical characteristics in the methylation score group. Including (A) Age, (B) Gender, (C) Treatment, and (D) Project. **Figure S4**. The discrepancies of risk score in different status of known biomarkers for glioma. (A) Kaplan‒Meier survival curves of GBM patients and LGG patients in the prognostic model. (B) MGMT status, IDH status and 1p19q status. **Figure S5**. Analysis of two key lncRNAs. (A) The key RNA methylation-related lncRNA associated regulatory network through LncBook database. (B) The survival analysis of high- and low-expression groups of RP11-98I9.4 and RP11-752G15.8. (C) the expression of two key lncRNAs in GBM and in LGG. **Figure S6**. The differences in IC50 between the two risk groups for the remaining ninety-seven small molecule compounds/drugs. **Figure S7**. GO and KEGG enrichment analysis of the predicted miRNA-mRNA of two key lncRNAs. (A ) Top significantly enriched GO terms of RP11-752G15.8. (B) KEGG pathway enrichment analysis of RP11-752G15.8. (C) Top significantly enriched GO terms of RP11-98I9.4. (D) KEGG pathway enrichment analysis of RP11-98I9.4.

**Additional file 2: Table S1**. The m6A/m5C/m1A score of TCGA glioma was calculated by GSVA.

**Additional file 3: Table S2**. M6A/m5C/m1A score-related lncRNAs were selected by WGCNA.

**Additional file 4: Table S3**. Differential expression analysis of common lncRNAs between glioma and normal tissues.

**Additional file 5: Table S4**. Twenty-six lncRNAs involved in m6A/m5C/m1A score-related lncRNAs and DE-lncRNAs.

**Additional file 6: Table S5**. The total enriched terms of GSEA GO analysis between the low- and high-risk groups.

**Additional file 7: Table S6**. The total enriched pathways of GSEA KEGG analysis between the low- and high-risk groups.

**Additional file 8: Table S7**. The three highest Pearson correlation results of immunoinfiltrated cells were associated with two RNA methylation-related lncRNAs.

**Additional file 9: Table S8**. The results of predict miRNA-mRNA (Table S8) for each key lncRNA by the miRTarBase database.

**Additional file 10: Table S9**. The results of GO terms and KEGG pathways enrichment analysis.

## Data Availability

All datasets involved in this study are included in the article or Additional files, and further inquiries can be directed to the corresponding author.
