# Supplementary material for: Using integrated analysis from multicentre studies to identify RNA methylation-related lncRNA risk stratification systems for glioma
Source: Cancer Cell Int. 2023 Aug 5;23:156. doi: 10.1186/s12935-023-03001-w (PMC10403900; doi:10.1186/s12935-023-03001-w)
Supplement: Supplementary file 1 — Additional file 1: Figure S1. Hierarchical clustering of the TCGA glioma samples. Figure S2. The TCGA cohort was classified into high- and low-scoring subgroups according to the m6A/m5C/m1A score of each type. Figure S3. Differences in clinical characteristics in the methylation score group. Including (A) Age, (B) Gender, (C) Treatment, and (D) Project. Figure S4. The discrepancies of risk score in different status of known biomarkers for glioma. (A) Kaplan‒Meier survival curves of GBM patients and LGG patients in the prognostic model. (B) MGMT status, IDH status and 1p19q status. Figure S5. Analysis of two key lncRNAs. (A) The key RNA methylation-related lncRNA associated regulatory network through LncBook database. (B) The survival analysis of high- and low-expression groups of RP11-98I9.4 and RP11-752G15.8. (C) the expression of two key lncRNAs in GBM and in LGG. Figure S6. The differences in IC50 between the two risk groups for the remaining ninety-seven small molecule compounds/drugs. Figure S7. GO and KEGG enrichment analysis of the predicted miRNA-mRNA of two key lncRNAs. (A ) Top significantly enriched GO terms of RP11-752G15.8. (B) KEGG pathway enrichment analysis of RP11-752G15.8. (C) Top significantly enriched GO terms of RP11-98I9.4. (D) KEGG pathway enrichment analysis of RP11-98I9.4. [file 12935_2023_3001_MOESM1_ESM.docx]

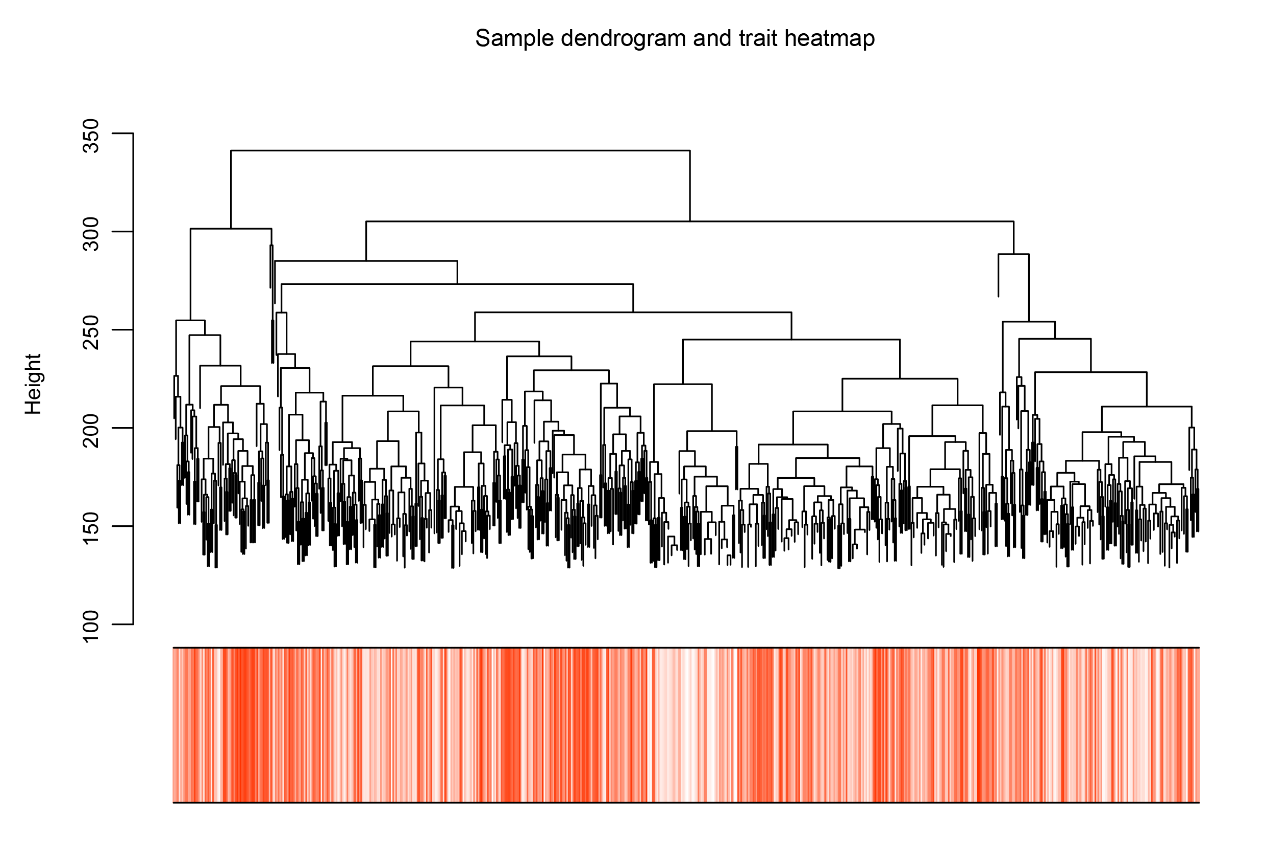


**Figure S1. Hierarchical clustering of the TCGA glioma samples.**


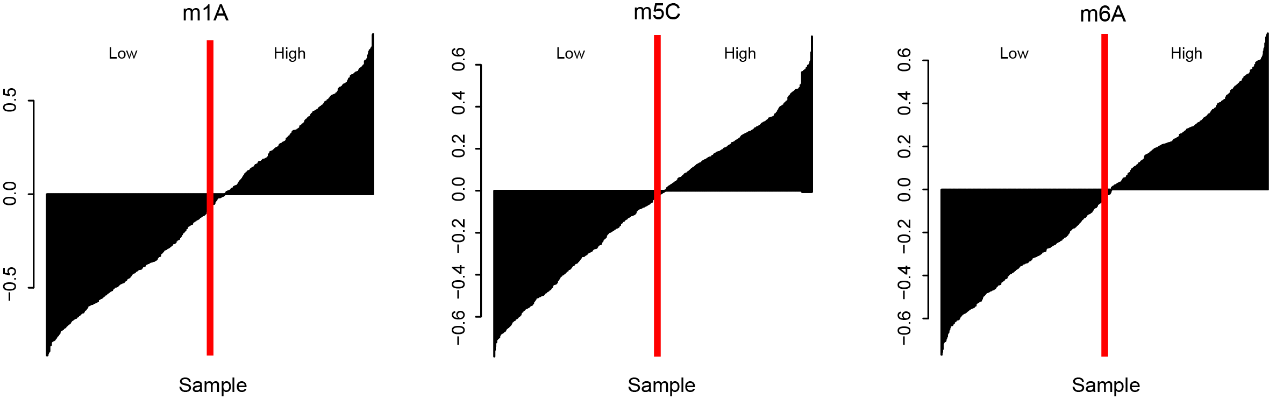


**Figure S2. The** **TCGA cohort was classified into high- and low-scoring subgroups according to the m6A/m5C/m1A score of each type.**


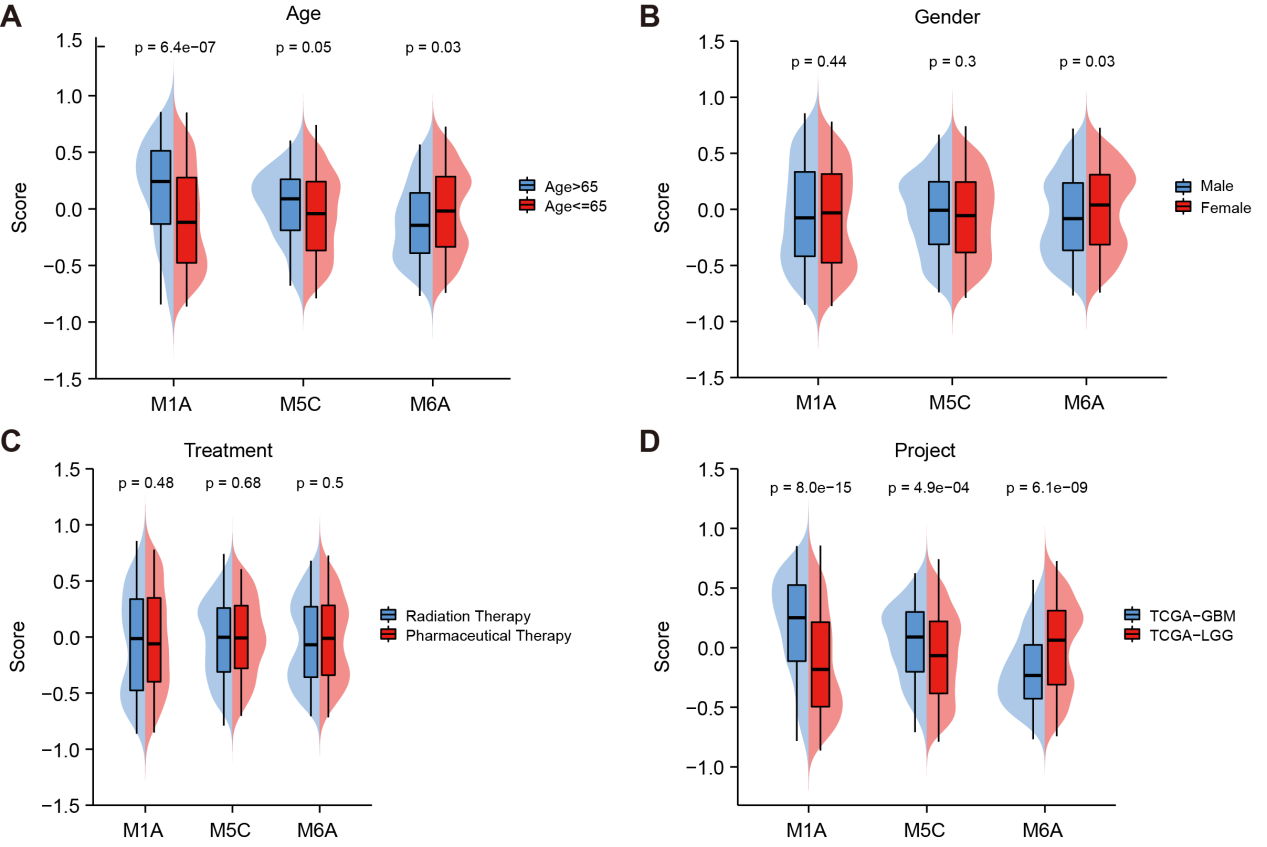


**Figure S3.** **Differences in clinical characteristics in the methylation score group. Including (A) Age, (B) Gender, (C) Treatment, and (D) Project.**


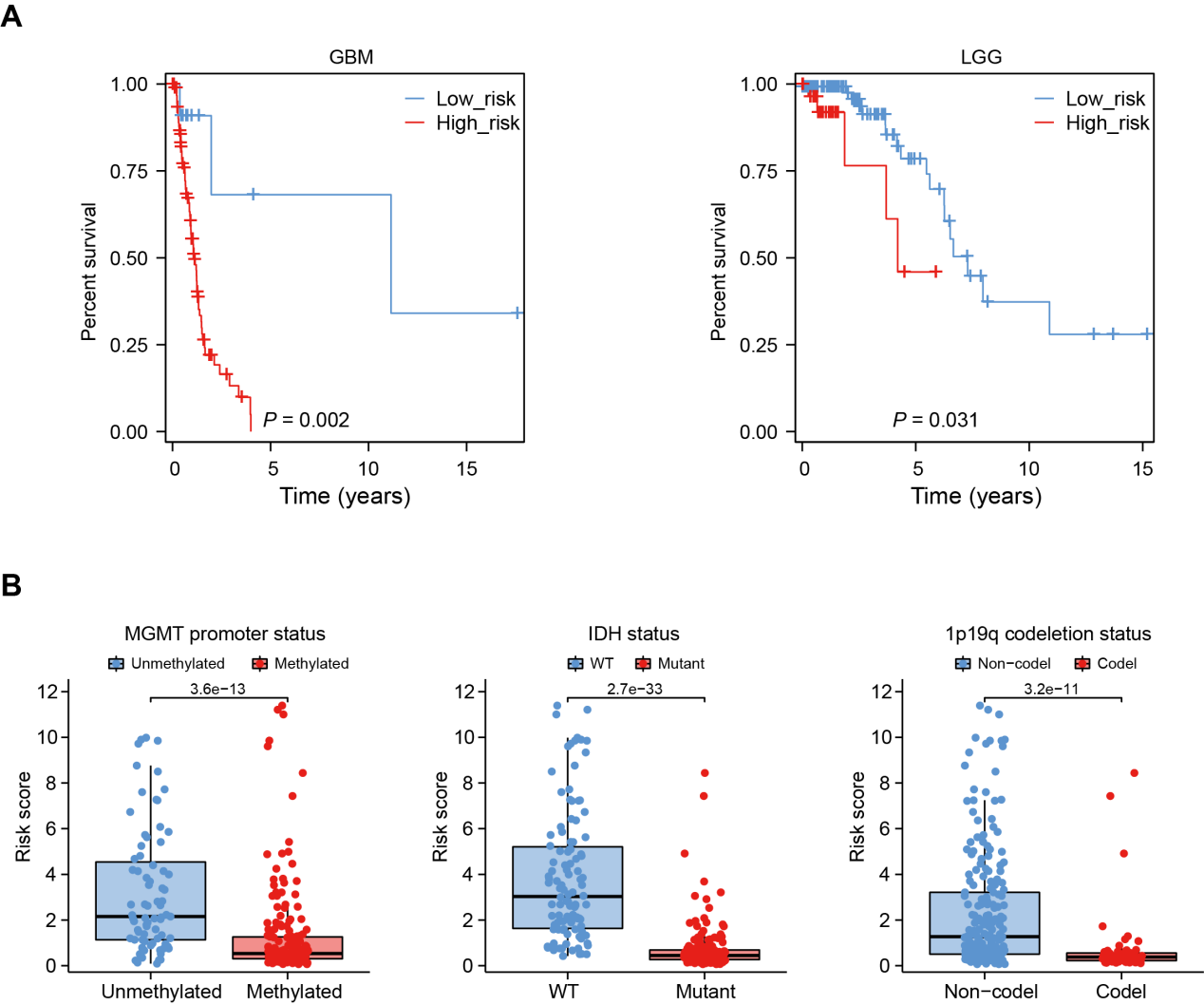


**Figure S4. The discrepancies of risk score in different status of known biomarkers for glioma. (A) Kaplan‒Meier survival curves of GBM patients and LGG patients in the prognostic model. (B) MGMT status, IDH status and 1p19q status.**


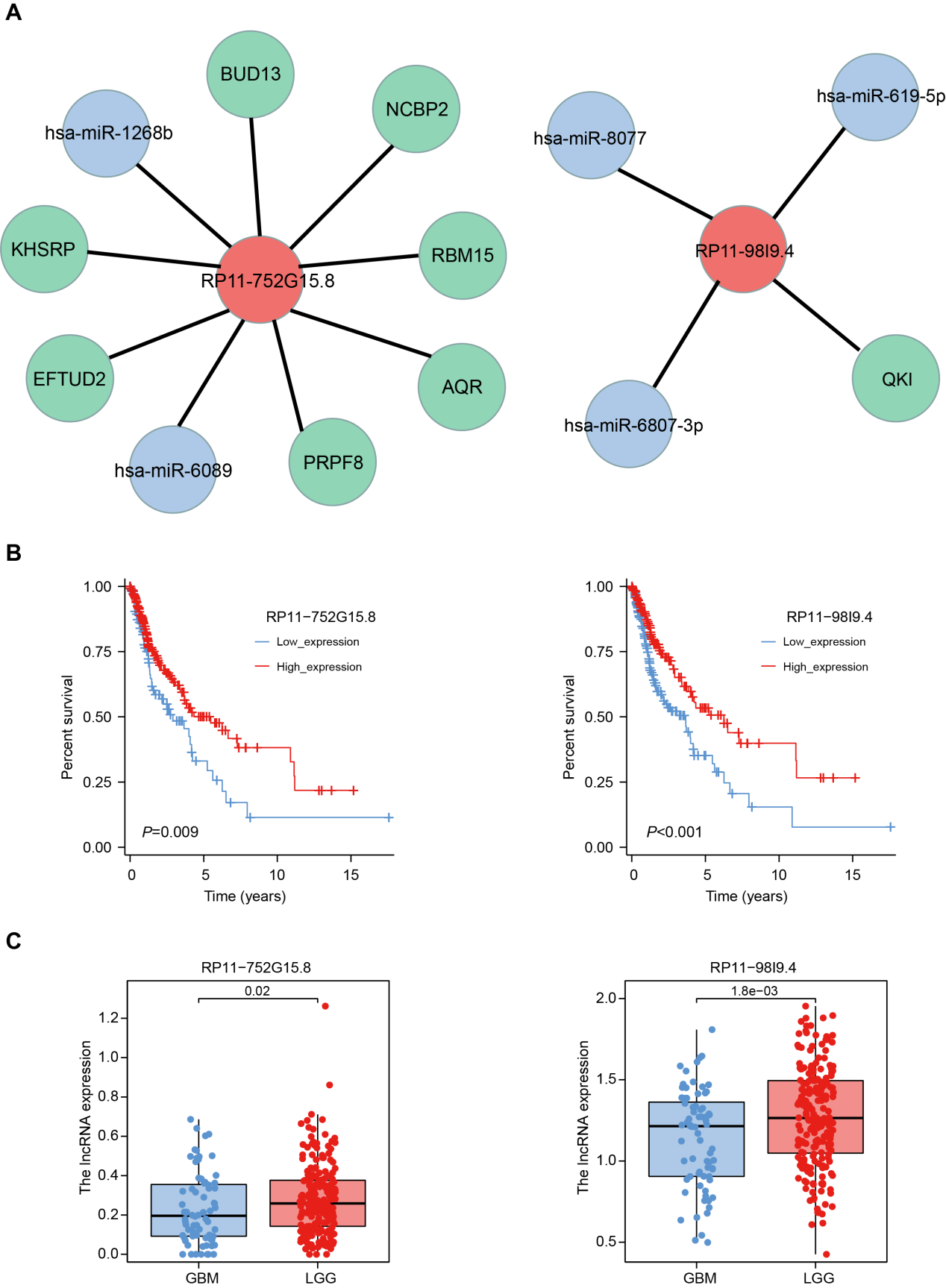


**Figure S5. Analysis of two key lncRNAs. (A) The key RNA methylation-related lncRNA associated regulatory network through LncBook database.** **(B) The survival analysis of high- and low-expression groups of RP11-98I9.4 and RP11-752G15.8. (C) the expression of two key lncRNAs in GBM and in LGG.**


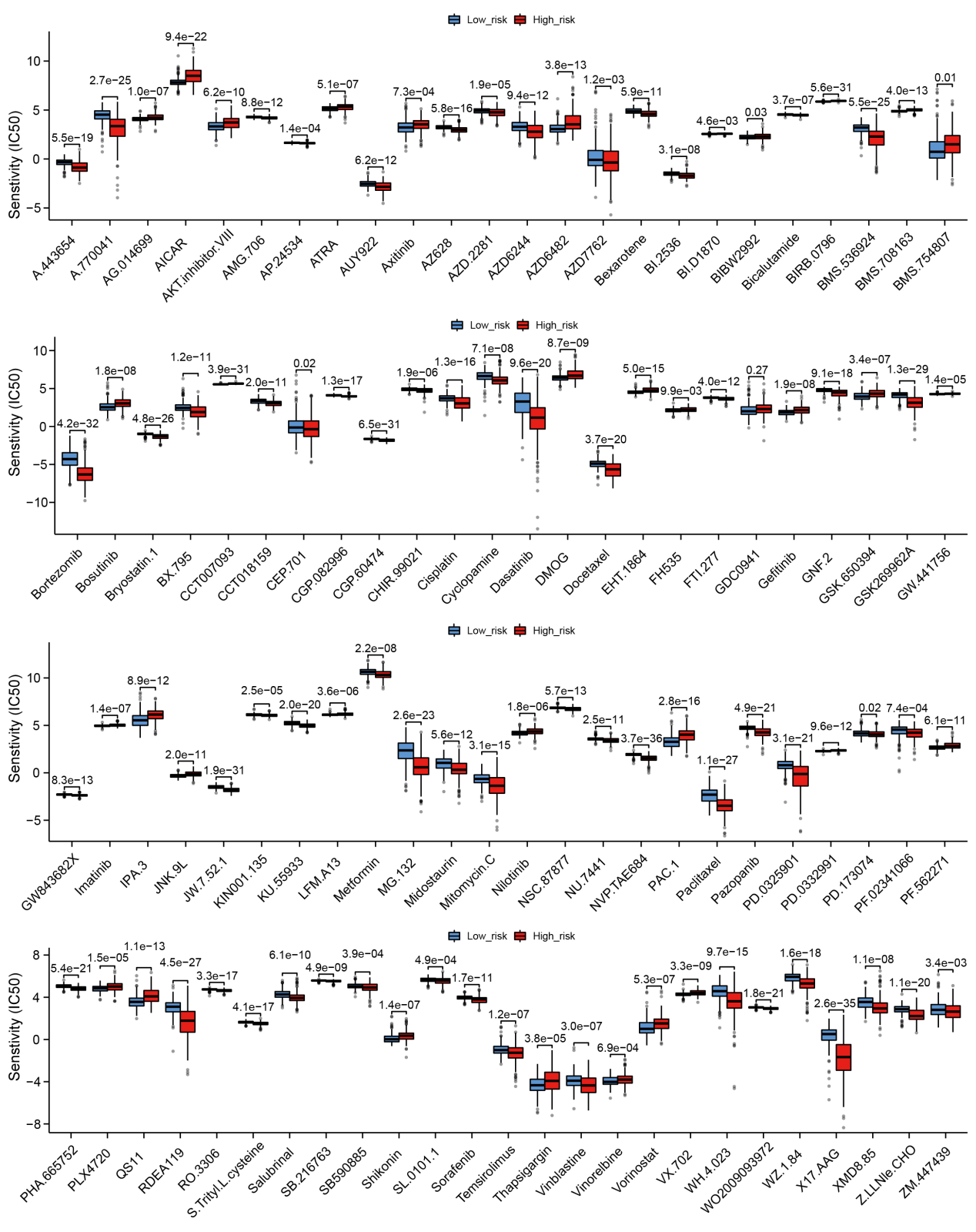


**Figure S6. The differences in IC50 between the** **two risk groups for the remaining ninety-seven small molecule compounds/drugs.**


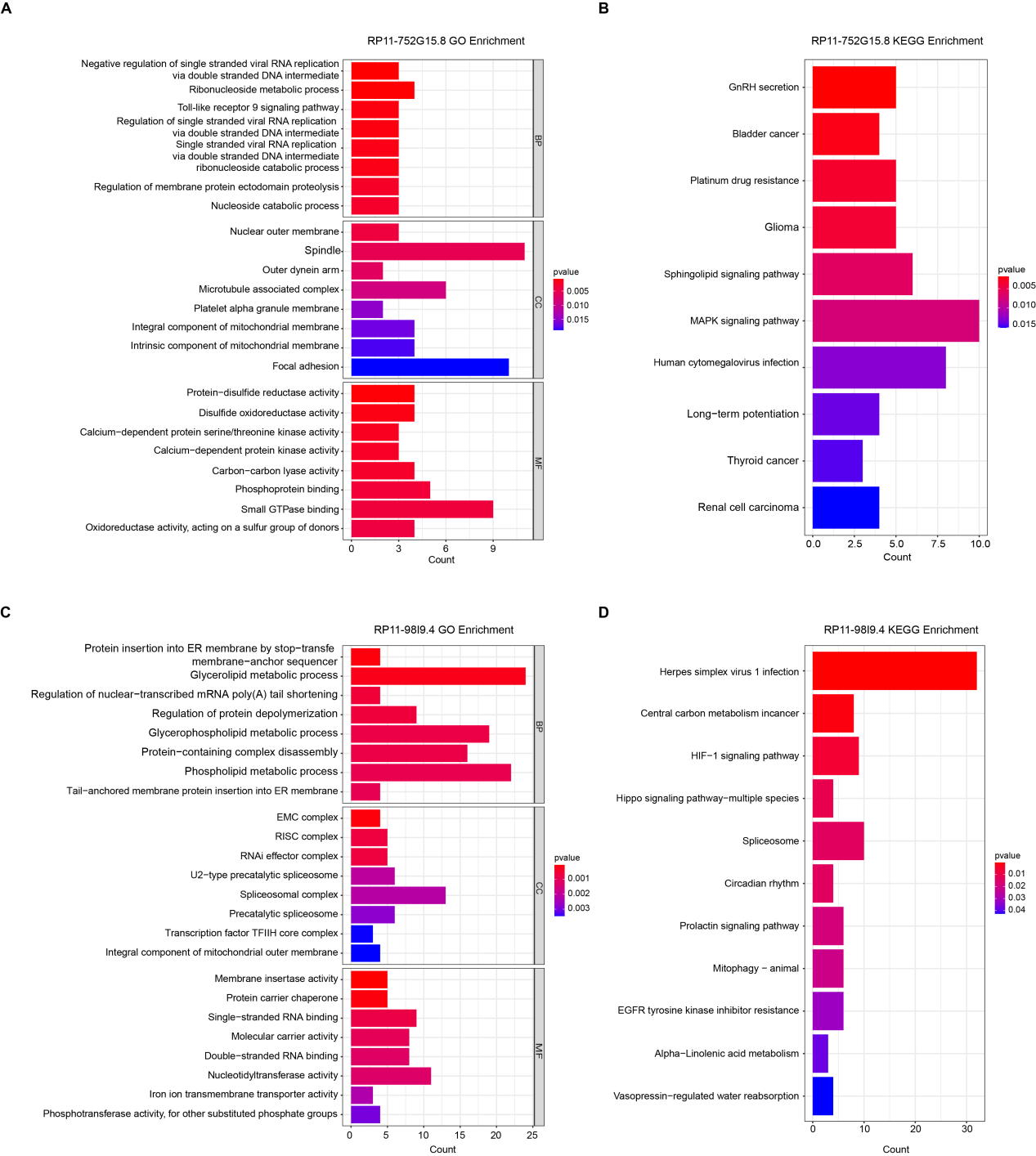


**Figure S7. GO and KEGG enrichment analysis of the predicted miRNA-mRNA of two key lncRNAs. (A ) Top significantly enriched GO terms of RP11-752G15.8. (B) KEGG pathway enrichment analysis of RP11-752G15.8. (C) Top significantly enriched GO terms of RP11-98I9.4. (D) KEGG pathway enrichment analysis of RP11-98I9.4.**
